# Clinicopathological and Epidemiological Findings in Pet Cats Naturally Infected with Feline Immunodeficiency Virus (FIV) in Australia

**DOI:** 10.3390/v14102177

**Published:** 2022-09-30

**Authors:** Caroline Carlton, Jacqueline M. Norris, Evelyn Hall, Michael P. Ward, Stephanie Blank, Shelby Gilmore, Anjuli Dabydeen, Vivian Tran, Mark E. Westman

**Affiliations:** 1 Lort Smith Anim al Hospital, 24 Villiers Street, North Melbourne, VIC 3051, Australia; 2 Sydney School of Veterinary Science, Faculty of Science, The University of Sydney, Sydney, NSW 2006, Australia; 3 The Sydney Institute for Infectious Diseases, The University of Sydney, Sydney, NSW 2006, Australia

**Keywords:** Australia, biochemistry, clinical signs, diagnosis, disease associations, FIV, hematology, survival time, urinalysis, veterinary science

## Abstract

Feline immunodeficiency virus (FIV) infection in experimentally infected domestic cats produces characteristic clinical manifestations including hematological changes, neurological disease, neoplasia (most notably lymphoma) and lymphopenia-mediated immunodeficiency predisposing cats to a range of secondary infections. Conflicting reports exist, however, with regard to disease associations and survival time in naturally FIV-infected cats. The purpose of this retrospective case–control study was to investigate the effect of natural FIV infection on hematological, blood biochemical and urinalysis parameters and survival time in three cohorts of pet cats in Australia. Cohorts 1 and 2 were recruited from a large veterinary hospital in Melbourne, Victoria (*n* = 525 and 282), while a third cohort consisted of cats recruited from around Australia as part of a FIV field vaccine efficacy trial (*n* = 425). FIV-infected cats in cohorts 1, 2 and 3 were found to have 15/37 (41%), 13/39 (33%) and 2/13 (15%) clinicopathological parameters significantly different to FIV-uninfected cats, respectively. Two changes in FIV-infected cats in cohort 1, hypochromia (low hemoglobin) and hyperglobulinemia, were outside the supplied reference intervals and should serve as diagnostic triggers for FIV testing. Kaplan–Meier survival analysis of cats in cohorts 1 and 2 combined did not find any difference between FIV-infected and FIV-uninfected cats, however a confounding factor was a large euthanasia rate within the first 12 months in both groups. Three significant (*p* < 0.05) spatial clusters of FIV infection were identified in Melbourne. A possible relationship between FIV infection status and socioeconomic disadvantage was discovered, based on three government indices of socioeconomic status (*p* < 0.001). Until longitudinal field studies are performed in Australia to further investigate the long-term effects of natural FIV infection, Australian veterinarians should consider FIV to be an important infection of pet cats, and recommend measures to prevent FIV infection.

## 1. Introduction

Feline immunodeficiency virus (FIV) is a retrovirus of the family Retroviridae which infects domestic and non-domestic felids. The virus was initially discovered in North America in 1986 in a colony of cats with signs of immunodeficiency [1], and has since been found globally [2,3]. In Australia, 15–16% of adult pet cats with outdoor access are FIV-infected [4,5]. Seven FIV subtypes are currently recognized (A to F and U-NZenv) [6,7,8,9], with no clear differences reported between subtypes with regard to pathogenesis [10]. Australian cats are predominantly infected with FIV-A, with occasional FIV-F and recombinant subtype A/B infections also reported [11,12,13].

FIV primarily infects mononuclear hematopoietic cells, including B lymphocytes, CD4+ T lymphocytes, CD8+ T lymphocytes, and monocytes/macrophages, although dendritic cells, microglia and astrocytes can also be affected [2,14]. Infection leads to a progressive decline in CD4+ lymphocytes, inversion of the CD4:CD8 ratio, and in some cases progression to severe immunodeficiency like that seen in humans infected with HIV [2,14,15,16,17]. Experimentally and naturally FIV-infected cats may show an increased susceptibility to opportunistic infections and immune dysregulation [15,18,19]. This can manifest clinically as oral cavity disease [4,20], renal disease [21,22], neurological disease and neurocognitive dysfunction [23,24,25], hematological abnormalities [1,19,26] and certain neoplasms [27,28]. In Australia, an association with FIV status has been found in cats with clinical signs of illness [5,29], and in cats presenting with periodontal disease, chronic kidney disease and lymphoma [21,30,31,32]. Other Australian studies, however, have reported no difference in FIV prevalence between ‘healthy’ and ‘sick’ cats [4,33].

Several studies have examined changes in hematological parameters due to experimental FIV infection. The seminal FIV paper reported transient neutropenia and leukopenia for up to four weeks post-inoculation, although these alterations were not consistently associated with the clinical status of the cats [1]. Other studies of experimentally FIV-infected cats have reported neutropenia, lymphopenia, eosinopenia and leukopenia, depending on the duration of FIV infection [34,35,36]. FIV has been detected within mononuclear cells and stromal cells in bone marrow aspirate samples from both experimentally and naturally infected cats, indicating the ability to infect myeloid progenitor cells and a potential inhibitory effect on hematopoiesis [37,38].

In regard to natural FIV infection, a large retrospective field study performed at a university hospital in Australia found FIV-infected cats were more likely to have low monocyte counts than FIV-uninfected cats, but no other significant differences in hematological or blood biochemical parameters were observed [39]. Another Australian study found naturally FIV-infected cats had significantly lower absolute numbers of neutrophils and total leukocytes than FIV-uninfected cats [40]. Other hematological alterations variably reported in naturally FIV-infected cats globally include neutropenia, lymphopenia, anemia, thrombocytopenia and pancytopenia [26,34,37,41]. One study in southern Brazil found FIV-infected cats were 19% more likely to be anemic compared to FIV-uninfected cats [42], while in contradistinction a study from northeastern Brazil did not demonstrate any hematological differences between FIV-infected and FIV-uninfected cats [43]. A study in Italy found shorter survival times in FIV-infected cats with anemia, but no effect on white blood cell indices [44].

Differences in blood biochemical parameters between FIV-infected and FIV-uninfected cats (whether experimentally or naturally infected) have been less frequently studied, with the only consistent finding being elevated globulin levels in FIV-infected cats, with or without an elevated total protein and a reduced albumin to globulin ratio [19,34,41,45]. The study from northeastern Brazil showed an increased risk of hyperbilirubinemia in FIV-infected cats, which had not been reported previously [43]. In Australia, one study of 326 cats recruited from throughout Western Australia reported an association between FIV status and hyperglobulinemia and azotemia, as well as lymphopenia [32,46].

Understanding the possible effects of FIV infection on hematological and blood biochemical measurements is important in understanding the pathogenesis of FIV in naturally infected cats, to assist clinicians with diagnostic triggers for FIV testing, and for prognostication purposes. The aim of the current study was to investigate the potential effects of natural FIV infection on hematology, blood biochemistry and urinalysis parameters and survival time in three cohorts of domestic pet cats (*Felis catus*) in Australia. The null hypothesis was that FIV-infected cats would have no clinicopathological changes compared to FIV-uninfected cats.

## 2. Materials and Methods

### 2.1. Study Populations—Cohorts 1 and 2

Cohorts 1 and 2 were comprised of a mixture of ‘apparently healthy’ and ‘sick’ client-owned and surrendered domestic cats presented to Lort Smith Animal Hospital in Melbourne, Victoria, Australia. The electronic medical database (Ezyvet Version 28.5, Auckland, New Zealand) was searched to identify cats tested for FIV between January 2013 and December 2019. In total, 3,021 patient histories were initially obtained for interrogation. Hematology, blood biochemistry and urinalysis results for cats with an FIV test result were matched with patient information obtained from the medical records. Cats with a FIV test result (whether positive or negative) but no accompanying hematology, blood biochemistry and urinalysis data were excluded. Cats with a FIV test result and incomplete hematology, blood biochemistry and/or urinalysis data were retained for analyses.

Cohort 1 consisted of cats with blood samples analysed by in-house pathology machines supplied by a commercial veterinary laboratory (IDEXX Laboratories Pty Ltd., Rydalmere, NSW, Australia). Cohort 2 consisted of cats with blood samples analysed at an external veterinary laboratory (IDEXX Laboratories, Mount Waverley, Victoria, Australia). The choice of analysis (i.e., in-house vs. external) was made by the attending veterinarian and included consideration of the cat’s clinical presentation and testing turnaround time (less than one hour for in-house analysis compared to up to 24 h for the external laboratory), the specific tests required, and in some cases financial constraints of the owner (external analysis was cheaper than in-house analysis). The analytes to be tested were determined by the attending veterinarian, and comprised standard panels offered by IDEXX Australia (https://www.idexx.com.au/en-au/, accessed on 31 December 2021).

### 2.2. Study Population—Cohort 3

Cohort 3 consisted of apparently healthy client-owned domestic cats recruited from five states/territories of Australia during 2013–2015 as part of a field efficacy study for a commercially available FIV vaccine (Fel-O-Vax^®^ FIV, Boehringer Ingelheim, Fort Dodge, IA, USA). Criteria for recruitment have been described [13]. Cats were recruited through participating veterinary clinics in Australia, most commonly at the same time as an annual health check or routine procedure (e.g., dental procedures). All cats were considered clinically healthy on physical examination by one of the authors (M.E.W.) at the time of testing. Hematology testing for cats in cohort 3 was performed by Veterinary Pathology and Diagnostic Services (VPDS), Sydney School of Veterinary Science (SSVS), the University of Sydney.

### 2.3. Testing Methods—Cohorts 1 and 2

Blood samples were collected by jugular, cephalic or lateral saphenous venipuncture and placed into sterile collection tubes. A small number of cats (13% in cohort 1, 14% in cohort 2) were sedated or anesthetised according to hospital protocol to facilitate restraint for blood collection (Table 1). When obtained, urine samples were collected via cystocentesis, as a voided sample, via a urinary catheter or by manual bladder expression. In-house blood and urine testing was generally performed within one hour of collection, while external blood testing was generally performed within 12 h of collection. If blood testing was not to be immediately performed, samples were refrigerated until required.

In-house hematology testing (cohort 1) was performed on ethylenediamine tetraacetic acid (EDTA) blood using a ProCyte Dx^®^ Hematology Analyser (IDEXX Laboratories). External hematology testing (cohort 2) was performed on EDTA blood using a Sysmex Xn^®^ Analyser (Sysmex, Kobe, Hyogo, Japan). Blood biochemical testing was performed on serum or plasma from lithium heparin, fluoride oxalate, gel tube or plain blood samples in-house (cohort 1) using a Catalyst Dx^®^ Chemistry Analyser (IDEXX Laboratories) or externally (cohort 2) using a Beckman Coulter 680^®^ Analyser (Brea, CA, USA). Reference intervals (RIs) for each analyte with both in-house and external testing were supplied by IDEXX Laboratories, Australia. 

For cats in both cohorts 1 and 2, point-of-care (PoC) testing on EDTA blood was performed in-house to detect anti-FIV antibodies and feline leukemia virus (FeLV) antigen, usually (but not always) on the same day as sample collection for hematology and blood biochemistry testing. Witness^®^ FIV/FeLV (Zoetis Animal Health, Lyon, France), SNAP^®^ FIV/FeLV Combo (IDEXX Laboratories, Westbrook, ME, USA) and Senspert^®^ FIV/FeLV (VetAll Laboratories, Gyeonggi-do, Korea) test kits were used. Witness^®^ FIV is an immunochromatographic test that detects antibodies against FIV envelope transmembrane glycoprotein gp40; SNAP^®^ FIV is an enzyme-linked immunosorbent assay (ELISA) that detects antibodies against FIV core proteins p15 and p24; and Senspert^®^ FIV is an immunochromatographic test that detects antibodies against gp40 and p24. All three PoC test kits detect FeLV capsid p27 antigen. Witness^®^ FIV is reported to have sensitivity and specificity of 100% and 100% in FIV-unvaccinated cats in Australia, and SNAP^®^ FIV 100% and 97%, respectively, [47]. Witness^®^ FeLV is reported to have sensitivity and specificity of 91% and 98% in cats in Australia, and SNAP^®^ FeLV 100% and 94%, respectively, [48]. The accuracy of the Senspert^®^ FIV/FeLV test kit in Australia has not been independently reported. A summary of the PoC test kits used for FIV testing in cohorts 1 and 2 is provided in Supplementary Table S1.

In some cases, residual EDTA blood was sent refrigerated for FIV molecular testing at a commercial laboratory (FIV RealPCR^TM^, IDEXX Laboratories, Brisbane, Queensland, Australia). FIV RealPCR^TM^ testing detects proviral DNA (FIV DNA inserted into the cat’s genome), as well as viral RNA, due to a reverse-transcriptase (RT) step that is performed as part of the PCR assay [49]. FIV RealPCR^TM^ is reported to have sensitivity and specificity of 92% and 99% in a population of FIV-vaccinated and FIV-unvaccinated cats in Australia [48].

Urinalysis testing was performed on cats in both cohorts 1 and 2 in-house using a VetLab UA^®^ Analyser (IDEXX Laboratories) for dipstick analysis, and a SediVue Dx^®^ Urine Sediment Analyser (IDEXX Laboratories) for urine sediment examination and urine specific gravity (USG) measurement.

### 2.4. Testing Methods (Cohort 3)

Blood was collected by jugular venipuncture and placed into two sterile EDTA tubes. One tube was transported refrigerated to VPDS for hematology testing using a Sysmex XT 2000i^®^ machine (Sysmex, Kobe, Hyogo, Japan) within 24 h of blood collection. Manual leukocyte differential counts were performed when the machine was unable to provide an automated differential count. If blood testing was not to be immediately performed, samples were refrigerated until required. RIs for each analyte were supplied by VPDS.

FIV/FeLV PoC testing was performed on all cats within 24 h of collection using Witness^®^ FIV/FeLV, SNAP^®^ FIV/FeLV Combo and Anigen Rapid^®^ FIV/FeLV (BioNote, Gyeonggi-do, Korea) test kits. Like Witness^®^ FIV/FeLV, Anigen Rapid^®^ FIV/FeLV tests detect antibodies against FIV gp40 and FeLV p27 antigen. Anigen Rapid^®^ FIV is reported to have sensitivity and specificity of 100% and 100% in FIV-unvaccinated cats in Australia [47]. Anigen Rapid^®^ FeLV is reported to have sensitivity and specificity of 91% and 98% in cats in Australia [48]. 

For all cats in cohort 3, the second tube of EDTA blood was sent refrigerated for FIV RealPCR^TM^ testing. In rare cases, additional blood was collected into a heparinised tube and sent refrigerated for virus isolation (VI) testing at one of two reference laboratories (Yamamoto Laboratory, The University of Florida, Gainesville, FL, USA; or Veterinary Diagnostic Services, The University of Glasgow, Scotland, UK) [47].

Blood biochemical testing was not performed, and urine not collected, from cats in cohort 3.

### 2.5. Case and Control Definitions (Cohorts 1 and 2)

A cat was defined as FIV-infected (‘case’) if it tested seropositive for FIV with PoC testing and had not been vaccinated against FIV based on the cat’s medical records and/or reported by the cat owner. A single positive FIV PoC test result was considered as a true positive and indicative of FIV infection. In some cases, the initial FIV PoC test result was confirmed by molecular testing (FIV RealPCR^TM^, IDEXX Laboratories), and/or by performing a second FIV PoC antibody test of the same or a different manufacturer, at the discretion of the attending clinician [48]. When a SNAP^®^ FIV test kit was used, a positive FIV result was interpreted with caution due to the known ability of the test to detect vaccinal antibodies [47,50]. If FIV vaccination status was unknown, a positive SNAP^®^ FIV result was always confirmed by FIV RealPCR^TM^ testing [47]. In two cases, (one from cohort 1, one from cohort 2), only FIV RealPCR^TM^ testing was performed (i.e., no FIV PoC antibody testing was done; Supplementary Table S1). A single positive FIV RealPCR^TM^ test result was also considered as a true positive and indicative of FIV infection.

A cat was defined as FIV-uninfected (‘control’) if it tested negative with FIV PoC testing.

Cats with discordant FIV test results (i.e., conflicting results with repeated FIV antibody testing, or a positive FIV PoC test result and a negative FIV RealPCR^TM^ result), or a positive FeLV PoC antigen test result, were excluded from the study.

### 2.6. Case and Control Definitions (Cohort 3)

Cats were defined as ‘cases’ (FIV-infected) and ‘controls’ (FIV-uninfected) using a published diagnostic algorithm due to a recent history (<12 months) of FIV vaccination in many cats [47]. This included consideration of all three FIV PoC test results, FIV RealPCR^TM^ testing, and, rarely, VI results. VI was performed on 17 occasions when there were discordant PoC and PCR results (*n* = 11), or to confirm FIV infection in FIV-vaccinated cats (*n* = 6) [47].

### 2.7. Data Collection (Cohorts 1 and 2)

Test results for each cohort were recorded in separate Microsoft Excel^®^ spreadsheets (Microsoft Office Professional Plus 2016, Version 2110, Redmond, WA, USA). Hematology and blood biochemistry results for cohort 1 and cohort 2 were analysed separately due to different machines being used for in-house and external testing, differences in test profiles (e.g., in-house testing measures 35–43 blood analytes, while the standard external laboratory feline profile measures 48 blood analytes), RIs provided specifically for each machine, and since results from different analysers cannot be directly compared [51,52,53,54].

Hematology results from a small number of cats were represented in both cohorts 1 and 2. In these 70 cases, external laboratory testing had been requested to confirm altered hematologic measurements reported with in-house testing. For these cats, results were included in both cohort 1 and 2. The in-house hematology results for duplicate cases were included as they were considered to possibly be a more accurate representation of red blood cell numbers and morphology due to the shorter time delay in sample processing (<1 h of sample collection), while the external hematology results for duplicate cases were retained as they were considered to possibly be an overall more accurate representation due to difficulties with the in-house analyser measuring white blood cells with altered morphology, most commonly band neutrophils [55].

Information obtained from medical records included age (years), breed (pedigree vs. non-pedigree), sex, neuter status (entire vs. neutered), date of FIV testing, FIV/FeLV PoC test kit used and result, relevant clinical examination findings to assist in determining health status, reason for FIV testing (wellness examination, hematological abnormality, dental disease, cat bite, systemic illness or dermatological disease; Table 2), date of blood and/or urine testing, whether the animal was hospitalized, and date of death or date of last visit to the hospital.

Health status (i.e., ‘healthy’ vs. ‘sick’) was retrospectively assigned by the primary author (C.C.) based on both the cat’s clinical condition at the time of testing, and the reason for testing, as previously described [21,25]. Cats were classified as ‘apparently healthy’ if FIV testing was performed as part of a routine health check, including admission to the hospital shelter, pre-anesthetic testing prior to sedation or general anesthesia for routine preventative procedures (e.g., neutering, treatment of cat fight wounds or dental disease). ‘Sick’ cats were those who presented for evaluation of a current disease process, and exhibited clinical signs of systemic illness (e.g., lethargy, anorexia, pyrexia, vomiting or diarrhea, weight loss, respiratory distress). Cats that presented with cat fight wounds, dental disease or dermatological disease were classified as ‘apparently healthy’ unless their clinical examination showed evidence of systemic illness (Table 2). 

The median time between FIV testing and hematology/blood biochemical testing was 0 days for cats in cohort 1 (range −12 days to 9 days; interquartile range [IQR] −1 to 0 days) and 1 day for cats in cohort 2 (range, −25 to 29 days; IQR 0–1 days). When more than one hematologic or blood biochemical test result was available for a cat, for the purposes of this study the data obtained closest to the date of FIV testing was used.

### 2.8. Data Collection (Cohort 3)

Test results were recorded in a Microsoft Excel^®^ spreadsheet. Information obtained from medical records included age (years), breed (pedigree vs. non-pedigree), sex, and neuter status (entire vs. neutered). The time between FIV testing and hematology testing was 0 days for all cats in cohort 3. Survival data were not collected for cats in cohort 3.

### 2.9. Statistical Analysis (Cohorts 1, 2 and 3)

Statistical analyses were performed using Genstat (v18, VSN International, Hemel Hempstead, UK). Analyses were conducted separately on the three cohorts. Age was analysed as both a continuous and categorical variable: Kitten (<1 year of age), Young Adult (1–5 years of age), Adult (5–10 years of age), Senior (10–15 years of age) and Geriatric (15+ years). Hematologic and blood biochemical data were assessed prior to analysis for normality using the Shapiro–Wilks test. Variables not meeting the requirements for normality were square root or log_e_ transformed as appropriate to approximate normality. Predicted means and standard errors for these variables were back-transformed as appropriate after analysis.

Descriptive analyses were conducted to determine the distribution of variables and their preliminary association with FIV status. To identify possible risk factors for FIV infection, logistic regression with an underlying binomial distribution was utilised to assess possible associations between outcome (FIV status) and factors including age (categorical), breed, neuter status, sex, health status and reason for FIV testing. To identify possible associations between FIV infection and hematology and blood biochemistry results, univariate analyses were initially performed on each hematological and blood biochemical variable using a REML (Reduced Maximum Likelihood) model. Factors analysed included FIV status, age (categorical and continuous), sex, neuter status, health status, breed and reason for testing (cats in cohort 3 had a reduced analysis since all were considered healthy at the time of testing and were only tested as part of a FIV vaccine field efficacy study). Any factor with *p* value < 0.25 was considered for inclusion in a multivariable model. A stepwise backwards elimination approach was used to obtain a final model for each outcome variable where all factors in the model were significant. Interactions were investigated within the final models. Post hoc Tukey’s tests to determine pairwise significance were performed for any category with greater than two levels. 

Variables with significant interactions were represented graphically as box and whisker plots using GraphPad Prism (Version 9.3.0, GraphPad Software, San Diego, CA, USA) with outliers retained.

Survival analysis using the Kaplan–Meier approach was conducted to compare survival times between FIV-infected and FIV-uninfected cats (calculated as date of death/censoring minus date of testing) by removing duplicate animals with results for both internal and external blood testing, and merging the remaining survival data for cohorts 1 and 2. Animals which had no recorded death date were censored. The log rank sum test was used for comparisons between groups.

For all analyses (except for univariate analysis), a *p* value < 0.05 was considered significant.

### 2.10. Mapping of Cases of FIV Infection and Assessment of Area-Based Socioeconomic Status (Cohorts 1 and 2 Only)

Data for cohorts 1 and 2 were combined and duplicate cases removed. Cases were mapped based on reported postcode of residence by joining these to a postcode shapefile of Australia (ArcGIS version 10.1 [ESRI, Redlands, CA, USA]). Potential geographic ‘hot spots’ of FIV infection, based on postcode longitude and latitude centroid coordinates, were investigated using the scan statistic SaTScan version 9.4.1 (Boston, MA, USA, www.satscan.org, accessed on 28 June 2022). A Bernoulli model (case–control) was used, with a circular scanning window of up to 50% of the population at risk, to identify clusters based on likelihood statistics (999 Monte Carlo simulations).

Socioeconomic data collected by the Australian government in the 2016 Census to develop the Socio-Economic Indexes For Areas (SEIFA) were analysed [56]. SEIFA consists of four indexes which describe different aspects of advantage and disadvantage (Index of Relative Socioeconomic Advantage and Disadvantage, IRSAD; Index of Relative Socioeconomic Disadvantage, IRSD; Index of Education and Occupation, IEO; and Index of Economic Resources, IER) and is a useful tool for interpreting area-based socioeconomic factors. Census data is used to assign all Australian postcodes a ranked decile (from 1 to 10) for the four SEIFA indexes, with a score of 1 representing the most socioeconomically disadvantaged areas and a score of 10 represents the least disadvantaged (i.e., most advantaged) areas. These postcode-based socioeconomic data were compared between FIV-infected and FIV-uninfected cats using Kruskal–Wallis one-way non-parametric ANOVAs (IBM SPSS Statistics 24.0).

Mapping and analysis of socioeconomic status of cases of FIV infection in cohort 3 was not performed due to a smaller sample size recruited from the entire country.

## 3. Results

### 3.1. Cohort 1—Study Population and Risk Factors for FIV Infection (In-House Testing; n = 525)

In-house hematology and/or blood biochemistry results were available for 539 cats. Twelve FIV-uninfected cats were excluded due to positive FeLV antigen results, and two cats excluded due to discordant FIV test results (positive antibody test and negative FIV RealPCR^TM^ result), leaving a sample size of 525 cats for cohort 1. 

Cohort 1 consisted of 324 males (62%; 67 entire, 257 neutered) and 201 females (38%; 18 entire, 183 neutered), with a median age of 6 years (range 2 months to 17 years, IQR 2.5–10 years), mostly of mixed breed (i.e., non-pedigree; 87%) (Table 1). Twelve kittens younger than 6 months-of-age were FIV tested, with only one 5-month-old kitten testing FIV-positive (Witness^®^ FIV test kit used, no PCR confirmation). All other kittens less than 6 months-of-age tested FIV-negative.

Hematology and biochemistry results were available for 436/525 (83%) cats, hematology only results were available for 19/525 (4%) cats, blood biochemistry only results were available for 48/525 (9%) cats, and urinalysis only results were available for 22/525 (4%) cats.

Urine biochemistry results were available for 134/525 (25%) cats, and urine specific gravity (USG) was available for 108/525 (20%) cats. Urine samples were collected via cystocentesis (58/134; 43%), as a voided sample (15/134; 11%), via a urinary catheter (9/134; 7%) or by manual bladder expression (8/134; 6%). The method of urine collection was not recorded for 44/134 (33%) cats.

Of the 525 cats, 133 (25%) cats were FIV-infected (105 [79%] male cats, including 29 entire and 76 neutered; 28 [21%] female cats, including 3 entire and 25 neutered). Confirmatory FIV RealPCR^TM^ testing was performed following a positive FIV PoC result in 17/133 cases, with all 17 cats testing PCR-positive for FIV subtype A.

Male cats were more than two times more likely to be FIV-infected than female cats (odds ratio [OR] 2.9, 95% confidence interval [CI] 1.9–4.7, *p* < 0.001). Entire cats were two times more likely to be FIV-infected than neutered cats (2.0, CI 1.2–3.3, *p* = 0.005). The median age of FIV-infected cats was 7.0 years (range 6 months to 17 years, IQR 4–10 years), and 5.5 years for FIV-uninfected cats (range 2 months to 17 years, IQR 2–10 years). The mean age of FIV-infected cats was higher than FIV-uninfected cats (*p* = 0.01; two-tailed *t*-test). Cats older than one year of age were more likely to be FIV-infected than kittens <1 year of age (OR 4.8, CI 1.1–20.9), with adult cats (5–10 years) and senior cats (10–15 years) almost eight times more likely to be FIV-infected than kittens (OR 7.9, CI 1.8–33.9 and OR 7.9, CI 1.8–34.4, respectively) (*p* = 0.02 for all). There was no association between breed and FIV status (*p* = 0.70).

At the time of FIV testing, 111 (21%) cats were classified as ‘healthy’ (29/133 [22%] of FIV-infected cats; 82/392 [21%] of FIV-uninfected cats) and 414 (79%) cats were classified as ‘sick’ (104/133 [78%] of FIV-infected cats; 310/392 [79%] of FIV-uninfected cats) by the attending veterinarian. There was no significant difference between FIV-infected and FIV-uninfected cats in terms of overall health status (i.e., ‘healthy’ vs. ‘sick’; *p* = 0.49).

Cats presenting with dental disease or cat fight wounds were more than four times more likely to be FIV-infected than cats presented for wellness examinations (dental disease - OR 4.2, CI 1.8–9.6, *p* < 0.001; cat fight wounds - OR 4.0, CI 1.6–10.3, *p* = 0.004). FIV infection was not associated with the presence of a hematological abnormality (anemia or leukopenia) or systemic illness (*p* = 0.87 and *p* = 0.48, respectively). Dermatological disease was excluded from analysis due to insufficient sample size (Table 3).

FIV-infected cats were not more likely to be hospitalized than FIV-uninfected cats (*p* = 0.61; Fisher’s exact test).

### 3.2. Cohort 2—Study Population and Risk Factors for FIV Infection (External Testing; n = 282)

External hematology and blood biochemistry results were available for 282 cats. No cats were FeLV-infected, and no cats were excluded due to discordant FIV results. 

Cohort 2 consisted of 170 males (60%; 28 entire, 142 neutered) and 112 females (40%; 8 entire, 104 neutered), with a median age of 6.4 years (range 2 months to 19 years, IQR 2.6–10.8 years), mostly of mixed breed (non-pedigree; 81%) (Table 1). Eleven kittens younger than 6 months-of-age were FIV tested, with all kittens testing FIV-negative.

Hematology and blood biochemistry results were available for 161/282 (57%) cats, hematology only results were available for 113/282 (40%) cats, blood biochemistry only results were available for 0/282 (0%) cats, and urinalysis only results were available for 8/282 (3%) cats.

Urinalysis results were available for only 16/282 (5%) cats, of which 8/16 (50%) were collected via cystocentesis and 1/16 (6%) was collected as a voided sample. The method of urine collection was not recorded for 7/16 (44%) cats. Urinalysis results were not analysed due to insufficient sample size.

Of the 282 cats, 61 (22%) cats were FIV-infected (51 [83%] male cats, including 35 entire and 16 neutered; 10 [17%] female cats, all neutered). Confirmatory FIV RealPCR^TM^ testing was performed following a positive FIV PoC result in 13/61 cases, with 10/13 cats testing PCR-positive for FIV subtype A. Subtyping results were not recorded for the other three cats.

Male cats were more than four times more likely to be FIV-infected than female cats (OR 4.4, CI 2.1–9.0, *p <* 0.001). Entire cats were more than three times more likely to be FIV-infected than neutered cats (OR 3.6, CI 1.7–7.4, *p* < 0.001). The median age of FIV-infected cats was 7.0 years (range 6 months to 17 years, IQR 4–10 years), vs. 6.0 years for FIV-uninfected cats (range 6 months to 19 years, IQR 2–11 years). The mean age of FIV-infected cats was not statistically different to FIV-uninfected cats (*p* = 0.12; two-tailed *t*-test). No association was found between FIV infection and age category (*p* = 0.13). Non-pedigree cats were three times more likely to be FIV-infected than pedigree cats (OR 3.0, CI 1.1–8.0, *p* = 0.012). 

At the time of FIV testing, 59 (21%) cats were classified as ‘healthy’ (13/61 [21%] of FIV-infected cats; 46/221 [21%] of FIV-uninfected cats) and 223 (79%) cats were classified as ‘sick’ (48/61 [77%] of FIV-infected cats; 175/221 [79%] of FIV-uninfected cats) by the attending veterinarian. Thus, similar to cohort 1, there was no significant difference between FIV-infected and FIV-uninfected cats in cohort 2 in terms of overall health status (i.e., ‘healthy’ vs. ‘sick’; *p* = 0.93).

Cats presenting with dental disease or cat fight wounds were more likely to be FIV-infected than cats presented for wellness examinations (dental disease—OR 5.8, CI 1.2–27.6, *p* = 0.013; cat fight wounds-OR 4.6, CI 1.4–15.6, *p =* 0.027). FIV infection was not associated with the presence of a hematological abnormality (anemia or leukopenia) or systemic illness (*p* = 0.93 and *p* = 0.53, respectively). Dermatological disease was excluded from analysis due to insufficient sample size (Table 3).

FIV-infected cats were not more likely to be hospitalized than FIV-uninfected cats (*p* = 0.90; Fisher’s exact test).

### 3.3. Cohort 3—Study Population and Risk Factors for FIV Infection (Fel-O-Vax^®^ FIV Field Efficacy Study; n = 425) 

Hematology results were available for 425 cats.

Cohort 3 consisted of 223 males (52%; 4 entire, 219 neutered) and 202 females (48%; all neutered), with a median age of 7.2 years (range 2.0 years to 20.2 years, IQR 5.3–10 years). The majority of cats were non-pedigree (370; 87%) (Table 1).

Of the 425 cats, 30 (7%) were FIV-infected (21 [70%] male cats, including 1 entire and 20 neutered; 9 [30%] female cats, all neutered). The median age of FIV-infected cats was 7.0 years (range 4 years to 16.1 years, IQR 5.5–9.4 years), vs. 7.2 years for FIV-uninfected cats (range 2 years to 20.2 years, IQR 5.2–10.1 years). The mean age of FIV-infected cats was not significantly different to FIV-uninfected cats (*p* = 0.95; two-tailed *t*-test).

No significant association was found between FIV infection and sex (*p* = 0.062), breed (*p* = 0.62) or age category (*p* = 0.71).

### 3.4. Cohort 1—Investigation of Possible Associations between FIV Status and Hematological, Blood Biochemical and Urinalysis Results (n = 525)

**Figure 1:**
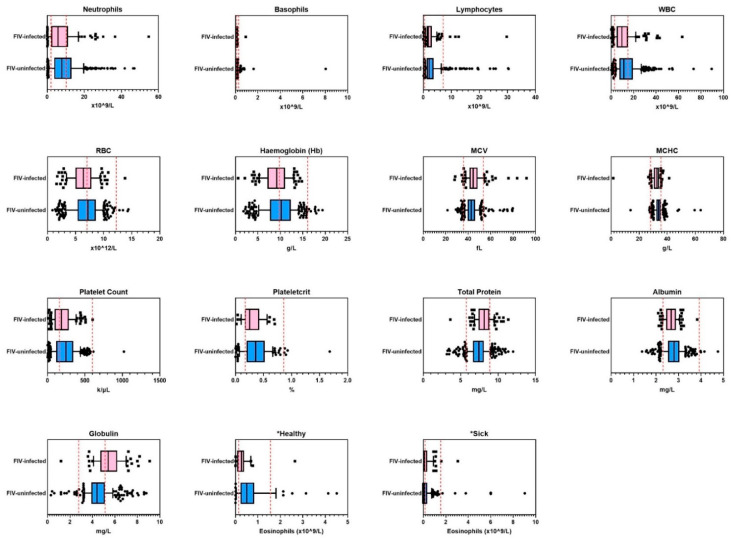
Hematology and blood biochemistry results in cohort 1 (*n* = 525; in-house hematology and blood biochemistry testing) from 15 of 37 measurements that were significantly different between FIV-infected and FIV-uninfected cats. Reference intervals are shown as vertical dashed red lines. WBC = white blood cell count, RBC = red blood cell count, MCV = mean corpuscular volume, MCHC = mean corpuscular hemoglobin concentration, HCT = hematocrit.

Following multivariate logistic regression analysis to compare results between FIV-infected and FIV-uninfected cats, FIV status remained significant in the final model for 15 of 37 measurements (Table 4, Figure 1). Of these 15 measurements, two differences were outside the provided RIs for normal healthy cats: mean hemoglobin was low in FIV-infected cats (hypochromia), and mean globulin concentration was high in FIV-infected cats. FIV-infected cats were also more likely to have both hypochromia and hyperglobulinemia when these variables were analysed together in a combined model. The risk of FIV infection increased when a cat had a low hemoglobin concentration (*p* = 0.024) and a high globulin concentration (*p* < 0.001).

Mean hemoglobin concentration was lower in hospitalized FIV-infected cats, when compared to FIV-infected cats that were not hospitalized (*p* = 0.049). Hemoglobin con-centration was below the RI for hospitalized FIV-infected cats, but within the RI for FIV-infected cats that were not hospitalized. When hospitalization was analysed in all cats, irrespective of FIV status, hospitalization did not affect mean hemoglobin concentration (*p* = 0.08). There was no significant difference in hemoglobin concentration when hospitalized FIV-infected cats were compared with hospitalized FIV-uninfected cats (*p* = 0.39).

Mean eosinophil concentration was lower in ‘healthy’ FIV-infected cats when compared to ‘healthy’ uninfected cats (*p* = 0.024), but this difference was not observed when ‘sick’ FIV-infected cats were compared to ‘sick’ uninfected cats (post hoc least significant difference testing) (Figure 1).

When results from FIV-infected and FIV-uninfected cats were compared as a binary outcome (e.g., eosinopenia YES/NO), four significant associations were found (Supplementary Table S2). FIV-infected cats were less likely to have lymphocytosis (OR 0.35, CI 0.1–1.0, *p* = 0.046), and more likely to have macrocytosis (OR 2.6, CI 1.3–5.4, *p* = 0.008), hyperproteinemia (OR 2.6, CI 1.5–4.5, *p* < 0.001), and hyperglobulinemia (OR 4.0, CI 2.6–6.1, *p* < 0.001) compared to FIV-uninfected cats. 

There were no significant interactions between FIV status and reason for testing in any of the hematological or blood biochemical parameters analysed. For example, FIV-infected cats with dental disease did not have higher mean globulin counts than FIV-uninfected cats with dental disease.

No significant differences in urinalysis results were found between FIV-infected and FIV-uninfected cats, although proteinuria did approach significance (*p* = 0.062).

### 3.5. Cohort 2—Investigation of Possible Associations between FIV Status and Hematological and Blood Biochemical Results (n = 282)

**Figure 2:**
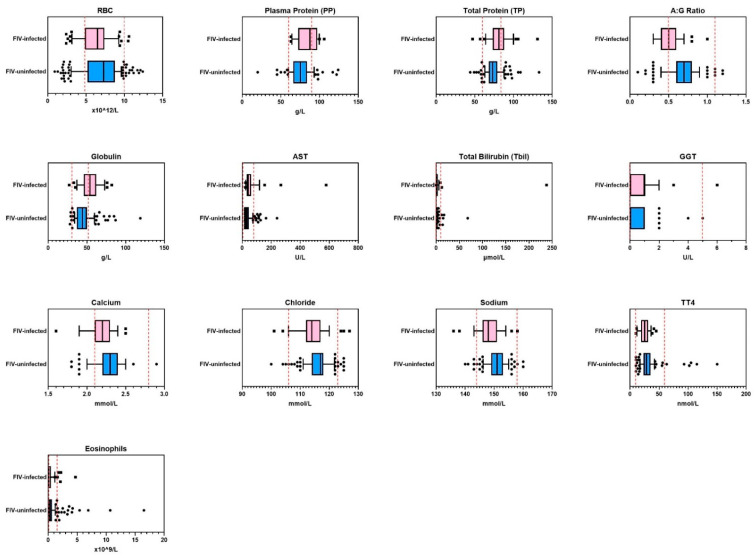
Hematology and blood biochemistry results in cohort 2 (*n* = 282; external hematology and blood biochemistry testing) from 13 of 39 measurements that were significantly different between FIV-infected and FIV-uninfected cats. Reference intervals are shown as vertical dashed red lines. HCT = hematocrit, RBC = red blood cell count, A:G ratio = albumin:globulin ratio, ALP = alkaline phosphatase, AST = aspartate aminotransferase, GGT = gamma glutamyl transferase, TT4 = thyroxine.

FIV status remained significant in the final multivariate logistic regression model for 13 of 39 measurements (Table 5, Figure 2). Of these 13 measurements, none were outside the provided RIs for normal healthy cats.

When results from FIV-infected and FIV-uninfected cats were compared as a binary outcome, two significant associations were found (Supplementary Table S3). FIV-infected cats were almost four times more likely to be hyperproteinemic (OR 3.7, CI 1.8–7.8, *p* < 0.001), and hyperglobulinemic (OR 3.9, CI 2.0–7.7, *p* < 0.001) than FIV-uninfected cats.

### 3.6. Cohort 3—Investigation of Possible Associations between FIV Status and Hematological Results (n = 425)

FIV status remained significant in the final multivariate logistic regression model for 2 of 13 hematological measurements, neither of which were outside the provided RIs for normal healthy cats. FIV-infected cats had a significantly lower hemoglobin concentration (FIV-infected 123.9 +/− 3.0 vs. FIV-uninfected 130.4 +/− 0.8; *p* = 0.041), and higher eosinophil counts (FIV-infected 0.575 +/− 0.02 vs. FIV-uninfected 0.407 +/− 0.06; *p* = 0.014) compared to FIV-uninfected cats (Table 6).

### 3.7. Survival Analysis (Cohort 1 and Cohort 2 Combined; n = 734)

After duplicate cases with results for both internal and external blood testing were removed, 184 FIV-infected cats and 550 FIV-uninfected cats with survival data remained from cohorts 1 and 2 for comparison. No survival data were available for cats in cohort 3.

**Figure 3:**
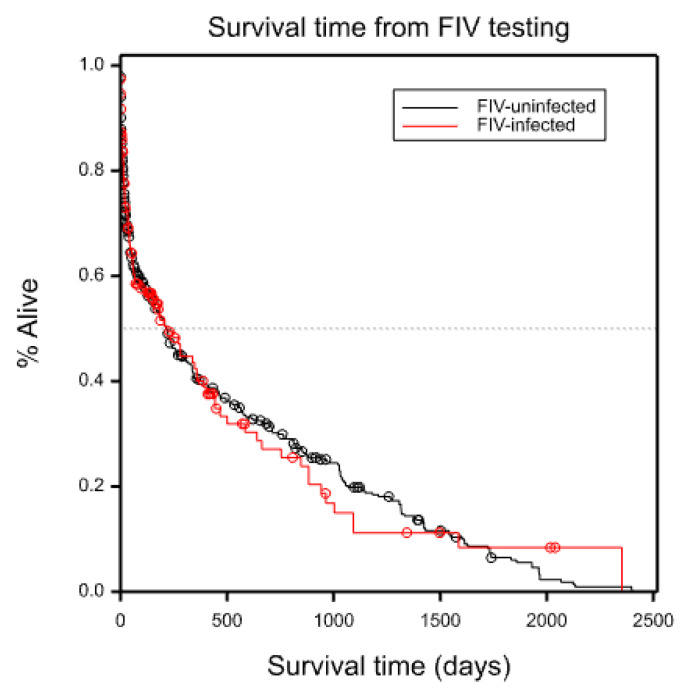
Kaplan–Meier survival analysis for cohorts 1 and 2 combined (*n* = 734). No significant difference in survival time was found between FIV-infected and FIV-uninfected cats (*p* = 0.97).

No significant difference in survival time was found between FIV-infected and FIV-uninfected cats in combined cohorts 1 and 2 (*p* = 0.97) (Figure 3).

In the combined cohort, 35 cats died during the study period (7 FIV-infected, 28 FIV-uninfected), and 224 cats were euthanased (80 FIV-infected, 144 FIV-uninfected). The cause of death was not recorded for 22 cats (6 FIV-infected, 16 FIV-uninfected). In total, 281/734 (38%) cats did not survive until the end of the study period.

The most common clinical conditions prompting euthanasia in both groups were concurrent hematological abnormalities, renal disease and neoplasia (Supplementary Table S4). Of the cats that did not survive due to neoplasia, lymphoma comprised 17/38 (45%), of which 7/17 (41%) were FIV-infected. FIV-infected cats were not more likely to present with lymphoma than FIV-uninfected cats (*p* = 0.75; Fisher’s exact test).

### 3.8. Mapping of Cases of FIV Infection and Assessment of Area-Based Socioeconomic Status (Cohorts 1 and 2 Combined; n = 734)

After duplicate cases with results for both internal and external blood testing were removed, 184 FIV-infected cats and 550 FIV-uninfected cats with postcode data remained from cohorts 1 and 2 for comparison (FIV prevalence 25%).

**Figure 4:**
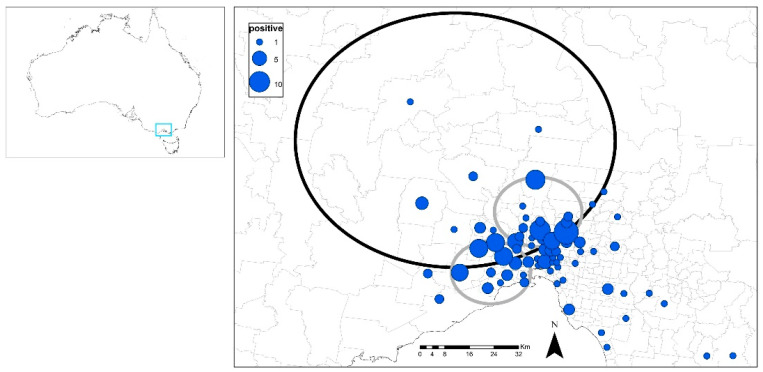
Cases of FIV infection in Melbourne, Victoria in cohorts 1 and 2 (*n* = 184), showing the significant clusters (black circle = primary, grey circles = secondary), and the number of cases per postcode as proportional symbols (blue dots). A map of Australia is included (left) to show the study area.

There was a primary cluster (northern edge of Melbourne; 37.445° S, 144.683° E, 41 km radius, 104 observed cases versus 72 expected cases) and two secondary, smaller (10–11 km radius) clusters (more within the central area of Melbourne). Overall, clustering was apparent in the northern and western areas of Melbourne (*p* < 0.05; Figure 4).

Three of the four SEIFA indexes (IRSAD, IRSD and IEO) were significantly lower for FIV-infected cats compared to FIV-uninfected cats (*p* < 0.001; Kruskal–Wallis one-way non-parametric ANOVA), demonstrating an association between FIV infection and socioeconomic disadvantage. Median values for FIV-infected and FIV-uninfected cats for IRSAD were 6.0 vs. 7.5. (i.e., FIV-infected cats 20% lower), for IRSD 5.0 vs. 7.0 (FIV-infected cats 29% lower), and for IEO 6.0 vs. 8.0 (FIV-infected cats 25% lower).

## 4. Discussion

Multiple studies have evaluated possible differences in clinicopathological results between naturally FIV-infected and FIV-uninfected cats, with variable and often inconsistent findings. In the current study, the largest field study performed to date in Australia to evaluate the possible effects of natural FIV infection on hematological, blood biochemical and/or urinalysis results, a plethora of subtle effects was observed. Although these alterations were not associated with a reduced survival time in cohorts 1 and 2, given the high euthanasia rate (224/734 [31%] in the combined cohort), as well as the high proportion of clinically unwell (‘sick’) cats (414/525 [79%] in cohort 1, 223/282 [79%] in cohort 2), the possibility that these changes may have a direct effect on both quality and quantity of life in FIV-infected cats requires further attention. It is also possible that the lack of association observed may be explained by geographic variations in FIV strain pathogenicity, mediated by genomic sequence differences that are yet to be identified [57]. Longitudinal studies investigating the clinical effects of FIV infection in different jurisdictions with varying FIV strains are required to help inform clinicians of the possible impacts of FIV infection on feline health.

Multiple significant differences in hematological and blood biochemical parameters were found between FIV-infected and FIV-uninfected cats. The majority of these differences were within the RIs for healthy cats supplied by the testing laboratory, and may have represented subclinical disease in FIV-infected cats that was not clinically apparent. Two important changes were observed in cohort 1 that were outside the supplied RIs and should serve as diagnostic triggers for veterinarians to perform FIV testing: low hemoglobin (hypochromia), even in the absence of concurrent anemia; and hyperglobulinemia, regardless of a cat’s health status or clinical signs. When results from FIV-infected and FIV-uninfected cats were compared as binary outcomes, FIV-infected cats in both cohorts 1 and 2 were significantly more likely to have hyperglobulinemia and hyperproteinemia than FIV-uninfected cats. Hyperglobulinemia and hypochromia were also significant in cohort 1 when analyzed in a combined model, with the risk of FIV infection increasing with increased globulin concentration and decreased hemoglobin concentration. This finding, to our knowledge, has not previously been reported. Hemoglobin was also lower in FIV-infected cats compared to FIV-uninfected cats in cohort 3, but the mean concentration remained within the RI.

A larger number of differences in hematological and blood biochemical parameters were observed between FIV-infected and FIV-uninfected cats in cohort 1 compared to cohort 2. Most notably, in cohort 2, the hemoglobin concentration and white blood cell counts of FIV-infected cats were not significantly different to those of FIV-uninfected cats (unlike in cohort 1). These discordant findings may be explained by differences in the severity of clinical illness in the two populations. Although the proportion of ‘sick’ cats did not differ between cohorts 1 and 2 (79% in both), the proportion of cats hospitalized in cohort 1 was significantly higher than the proportion of cats hospitalized in cohort 2 (311/525 [59%] vs. 138/282 [49%]; *p* = 0.006; Fisher’s exact test). Due to the retrospective nature of the study, hospitalisation data were analysed in lieu of clinical scoring to estimate the severity of illness. It is reasonable to surmise that cats who required hospitalization for diagnostic investigation and/or treatment of their illness were exhibiting more severe clinical signs than cats who were able to be treated as hospital outpatients.

The possible mechanism for FIV-induced hypochromia is currently unknown. No studies to date have documented FIV infection of cells of the erythroid lineage. An indirect inhibitory effect on hematopoiesis has been proposed through alterations in cytokine profiles and concentrations within the bone marrow, possibly induced via infection of stromal cells [37]. Alternatively, the possibility that a systemic pro-inflammatory state may be induced by increased production of specific cytokines (e.g., interleukin-1, interleukin-6, tumor necrosis factor) which has been documented in FIV-infected cats [58], should be considered. Cytokine-mediated changes in iron availability, such as iron retention within macrophages, are common in people diagnosed with anemia of inflammatory disease (AID) [59] and could contribute to the tendency towards hypochromia in FIV-infected cats. The presence of lower mean hemoglobin in FIV-infected cats in cohort 3, although within the RI, supports the idea of a pro-inflammatory state, as these cats were apparently healthy. The findings of lower mean albumin (cohort 1) and lower mean thyroxine (cohort 2) levels in FIV-infected cats compared to FIV-uninfected cats also supports the theory of FIV infection causing a systemic pro-inflammatory state and non-thyroidal illness, with albumin having a role as a negative acute phase protein (decreased levels in response to systemic inflammation) [60]. In addition, the increased risk of FIV infection found in cats with concurrent hypochromia and hyperglobulinemia is consistent with chronic antigenic stimulation and subsequent predisposition to AID.

Hyperglobulinemia is a common clinicopathological finding in humans infected with HIV, and has been described as a hallmark of HIV infection [61]. A similar association between FIV infection and an elevated globulin count has been commonly reported in cats [19,26,41,45]. In both people and cats, hyperglobulinemia is attributed to increased antibody production by B cells (known as B cell expansion), triggered by direct and indirect viral effects, including altered cytokine production and activation of specific T cell populations (e.g., T helper cells) [62]. The result is a polyclonal gammopathy, an elevated level of circulating immunoglobulins which do not contribute to an effective immune response. Globulin levels can also be affected by breed, with a recent study reporting that healthy Ragdoll cats sampled in Italy had lower mean total protein compared to healthy non-Ragdoll cats [63].

Sex was found to affect globulin levels in the current study, with male cats regardless of FIV status having significantly higher mean total protein and globulin levels compared to female cats (cohorts 1 and 2). Due to the higher proportion of male cats in the FIV-infected groups (cohort 1—105/133 [79%], cohort 2—51/61 [84%]) compared to females, this potential sex effect must be considered when evaluating the effect of FIV infection on globulin levels. The risk and magnitude of hyperglobulinemia may be less pronounced with FIV infection than with HIV infection, especially in female cats. Nevertheless, the finding of higher mean globulin in FIV-infected cats, independent of sex, is in agreement with other studies [19,34,41,45].

The majority of cats in cohorts 1 and 2 was comprised of naturally infected cats from Melbourne, Australia, a large city with a reported FIV prevalence of approximately 15% [24]. Cats were either owned or surrendered by the general public to a large centrally located animal shelter and veterinary hospital, and presented for health checks or evaluation of a current illness. The FIV seroprevalences reported in this study (24% for cohort 1, 22% for cohort 2, 25% combined overall for cohorts 1 and 2) were therefore much higher than that previously reported for Melbourne. Possible reasons for this discrepancy include a higher proportion of surrendered or stray cats, and the large geographical area serviced by the hospital, which encompasses metropolitan Melbourne and surrounding regional areas. Differences in cat owner demographics may also be a factor, as the hospital provides financial assistance for owners unable to afford treatment for their pets, and socioeconomic status has been shown to be associated with FIV prevalence [56]. The FIV prevalence of pet cats in cohort 3 (7%), recruited from a number of clinics across the country, was lower than previously reported in cats with outdoor access in Australia [4,5]. The difference in FIV prevalence between the three cohorts may have represented geographic differences in Australia relating to FIV infection, or possibly lifestyle risk factors for FIV infection such as the amount of outdoor access permitted by owners [64]. 

As reported previously in Australia, cats with FIV infection in cohorts 1 and 2 were significantly more likely to reside in disadvantaged areas (lower socioeconomic scores) than advantaged areas (higher socioeconomic scores). Three of four SEIFA indexes were found to be 20–29% lower for FIV-infected cats compared to FIV-uninfected cats, supporting a likely relationship between socioeconomic status and feline health [56]. Interestingly, in both the previous report and the current study, the only SEIFA index not found to be associated with FIV status was the Index of Economic Resources (IER), suggesting a lack of education may be more of a factor than economic limitations. This should encourage Australian veterinarians to continue to spend time educating cat owners about measures for FIV prevention. Since detailed information regarding husbandry was not available for any of the recruited cats, further speculation about the role of lifestyle risk factors was not possible.

FIV-infected cats had lower mean total thyroxine (TT4) concentration compared to uninfected cats (cohort 2). To our knowledge, reduced TT4 concentrations in FIV-infected cats in comparison to a control (FIV-uninfected) group has not been previously reported. One study showed a lower thyroxine level in a cohort of untreated FIV-infected cats in comparison to a cohort of FIV-infected cats treated with zidovudine, but that study did not include FIV-uninfected cats [65]. Non-thyroidal illness syndrome (NTIS) is a well-established phenomenon in human and veterinary medicine and is thought to be an adaptive response which attempts to decrease energy consumption and metabolic demands during an active illness [66]. Animals with NTIS show alterations in thyroid hormone measurements, most commonly a low tri-iodothyronine (T3) level with acute illness, and a tendency towards low thyroxine (TT4) with prolonged and/or severe illness. In FIV-infected cats, lower TT4 may be attributed to the presence of a concurrent illness that is unrelated to retroviral status, or an indirect viral effect on thyroxine production (e.g., cyttokine-mediated). A similar effect on thyroxine levels is seen in people infected with HIV, with subclinical hypothyroidism considered common [67].

Several limitations were identified in this study. Due to the retrospective nature of the study, FIV infection was not confirmed in every case of a positive PoC test result in cohorts 1 and 2 by either performing a second PoC test kit or PCR testing, as per major feline retroviral testing guidelines [3]. Consequently, some false-positive FIV test results may have led to incorrect classification according to FIV status, including one 5-month-old kitten that may have had maternal antibodies present (FIV antibody testing in kittens is usually recommended from 6 months-of-age) [3,68]. In most cases in cohorts 1 and 2, prior FIV vaccination history was not known, and therefore inclusion of a small number of uninfected FIV-vaccinated cats in the FIV-infected group cannot be ruled out [47]. Disease causation was not able to be demonstrated and instead our study was only able to identify possible associations between FIV status and clinicopathological abnormalities. Complete hematological, blood biochemical and urinalysis results were not available for every cat, and the missing data may have contributed to statistical biases. The method of case selection, (i.e., cats with an FIV test result and clinicopathological test results) will have influenced the population under investigation, and led to a high proportion of ‘sick’ cats in the study population, thereby introducing some biases. The clinicopathological tests performed were determined by the attending veterinarian, and thus were based on an individual cat’s signalment and clinical signs, as well as the veterinarian’s knowledge and expertise. For cohort 1 and 2, due to the relatively high hospital case load and large veterinary team, it is likely that differences in veterinarians’ knowledge and experience of the potential impact of FIV significantly influenced the availability of results. As the study period for these cohorts encompassed approximately six years (2013–2019), it is also likely that emerging research regarding FIV during this time also affected veterinarians’ inclination to request retroviral testing. This influence may not be apparent in cohort 3, where cats were recruited at the time of routine veterinary intervention (e.g., vaccination, dental procedure) and hematology results were available for all cats. An underrepresentation of clinically healthy cats in cohorts 1 and 2 may have concealed the true effect of FIV infection on lifespan. Interpretation of survival analysis data was also affected by an inability to determine the duration of FIV infection for cats in the current study. Since increased duration of FIV infection is associated with increased viral genetic mutation and changes in cell tropism [69], cats with more chronic infections may have presented with more severe and/or different illnesses, and this may have influenced their clinical presentation and clinicopathological measurements.

## 5. Conclusions

Multiple clinicopathological differences were seen between naturally FIV-infected and FIV-uninfected cats in Australia. In particular, the presence of hypochromia and/or hyperglobulinemia should alert clinicians to the possibility of FIV infection. The wide range of clinicopathological changes observed in FIV-infected cats, despite generally being subtle and within RIs, may affect quality and/or quantity of life. Although a direct effect of FIV infection on quality of life and lifespan could not be demonstrated, the clinicopathological changes identified highlight that prospective longitudinal field studies are needed to investigate possible long-term effects of natural FIV infection on morbidity and mortality in the field, and possible variations in pathogenicity between FIV field isolates and geographical location.

## Figures and Tables

**Table 1:** Signalment details for cohort 1 (*n* = 525; in-house hematology and blood biochemistry testing), cohort 2 (*n* = 282; external hematology and blood biochemistry testing) and cohort 3 (*n* = 425; FIV vaccine efficacy study). ME = male entire, MN = male neutered, FE = female entire, FN = female neutered. Wellness = wellness examination, Cat bite = cat fight wounds, Dental = dental disease, Hem = hematological abnormality, Unwell = systemic illness, Derm = dermatological disease (refer to Table 2 for a complete description of these categories). FIV vacc = FIV vaccine efficacy field study. NA = not available.

Parameter		Cohort 1			Cohort 2			Cohort 3		
		FIV −	FIV +	Total	FIV −	FIV +	Total	FIV −	FIV +	Total
Age group (years)	<1 1 to 5 5–10 10 to 15 15+	36 165 115 68 8	2 44 61 25 1	38 209 176 93 9	19 84 62 49 7	3 19 28 10 1	22 103 90 59 8	0 126 188 72 9	0 10 16 3 1	0 136 204 75 10
Sex/neuter status	ME MN FE FN	38 181 15 158	29 76 3 25	67 257 18 183	12 107 8 94	16 35 0 10	28 142 8 104	3 199 0 193	1 20 0 9	4 219 0 202
Breed	Non-pedigree Pedigree	331 61	125 8	456 69	174 47	56 5	230 52	343 52	27 3	370 55
Health status	Healthy Sick	82 310	29 104	111 414	46 175	13 48	59 223	395 0	30 0	425 0
Sedation/ anesthesia	Yes ^1^ No	52 340	15 118	67 458	30 191	9 52	39 243	0 395	0 30	0 425
Reason for testing	Wellness	53	11	64	29	5	34			
	Cat bite	18	15	33	5	5	10			
	Dental	31	27	58	15	12	27			
	Hem	49	11	60	44	8	52			
	Unwell	227	61	288	121	29	150			
	Derm	14	8	22	7	2	9			
	FIV vacc	0	0	0	0	0	0	395	30	425
Hospitalisation	Yes No	235 157	76 57	311 214	109 112	29 32	138 144	NA	NA	NA
TOTAL		392	133	525	221	61	282	395	30	425

^1^ If administered, sedative drugs were used at standard doses based on hospital protocol: alfaxalone 0.5–5 mg/kg, acepromazine 0.03 mg/kg, methadone 0.2–0.4 mg/kg, medetomidine 8–20 ug/kg, buprenorphine 0.01–0.02 mg/kg, butorphanol 0.2 mg/kg, Zoletil^®^ (tiletamine/zolazepam) 3–5 mg/kg, ketamine 5–10 mg/kg, diazepam 0.2–0.5 mg/kg, and gabapentin 5–10 mg/kg.

**Table 2:** Reasons for FIV testing in cohorts 1 and 2. Health status (i.e., ‘healthy’ vs. ‘sick’) was retrospectively assigned by the primary author (C.C.).

Reason for FIV Testing	Description	‘Healthy’ vs. ’Sick’
Wellness examination	Cats tested as part of a wellness examination, including surrendered cats tested before admission to the shelter	Healthy
Hematological abnormality	Cats with anemia or leukopenia where these results were a trigger for FIV testing	Sick
Dental disease	Cats presenting with severe periodontal disease and/or evidence of gingivostomatitis	Healthy *
Cat bite	Cats presenting with cat fight wounds (e.g., puncture marks in the skin, cellulitis, abscess, pyrexia)	Healthy *
Systemic illness	Cats presenting with non-specific clinical signs (e.g., lethargy, anorexia, pyrexia), gastrointestinal signs (vomiting or diarrhea), neurological signs (e.g., seizures, ataxia), or respiratory signs (e.g., tachypnea, respiratory distress, pyothorax)	Sick
Dermatological disease	Cats presenting with primary dermatological disease (e.g., pruritus, alopecia, pyoderma) or poor wound healing	Healthy *

* Cats tested due to dental disease, cat fight wounds and dermatological disease were assigned as ‘healthy’ unless their clinical examination showed evidence of systemic illness.

**Table 3:** Analysis of possible risk factors for FIV infection in cohort 1 (*n* = 525; in-house hematology and blood biochemistry testing), cohort 2 (*n* = 282; external hematology and blood biochemistry testing) and cohort 3 (*n* = 425; FIV vaccine efficacy study). OR = odds ratio, LCI = lower confidence interval, UCI = upper confidence interval, kitten = <1 year of age, young adult = 1–5 years of age, adult = 5–10 years of age, senior = 10–15 years of age, geriatric = 15+ years. Wellness = wellness examination, Cat bite = cat fight wounds, Dental = dental disease, Hem = hematological abnormality, Unwell = systemic illness (refer to Table 2 for a complete description of the ‘Reason for FIV testing’ categories). NA = not applicable.

Category	Cohort 1					Cohort 2					Cohort 3				
	% FIV-Infected	OR	LCI	UCI	*p* Value	% FIV-Infected	OR	LCI	UCI	*p* Value	% FIV-Infected	OR	LCI	UCI	*p* Value
Healthy Sick	27.7 24.6	0.85	0.54	1.4	0.49	22.0 21.5	0.97	0.49	1.9	0.93	7.1 NA	NA	NA	NA	NA
Non-pedigree Pedigree	24.2 25.8	1.1	0.71	1.7	0.70	9.6 24.3	3.0	1.2	8.0	0.012	7.3 5.45	0.73	0.22	2.5	0.62
Neutered Entire	22.9 37.6	2.0	1.2	3.3	0.005	18.3 44.4	3.6	1.7	7.4	<0.001	6.9 25.0	4.5	0.46	44.4	0.20
Female Male	13.9 32.4	3.0	1.9	4.7	<0.001	8.9 30.0	4.4	2.1	9.1	<0.001	4.5 9.1	2.2	0.96	4.8	0.062
<1	5.3	-	-	-	0.02 ^1^	13.6	-	-	-	0.13 ^1^	NA				
1 to 5	21.3	4.8	1.1	20.9		18.5	1.4	0.38	5.3		5.7	-	-	-	0.71 ^2^
5–10	30.5	7.9	1.8	33.9		31.1	2.9	0.78	10.5		8.4	1.5	0.55	4.2	
10 to 15	30.4	7.9	1.8	34.4		16.9	1.3	0.32	5.2		5.2	0.90	0.25	3.2	
15+	29.2	7.4	1.4	39.3		12.5	0.90	0.08	10.2		7.7	1.4	0.15	12.9	
Wellness	17.2				<0.001 ^3^	14.7				0.007 ^3^	NA	NA	NA	NA	NA
Cat Bite	45.5	4.0	1.6	10.3		50.0	5.8	1.2	27.6						
Dental	46.6	4.2	1.8	9.6		44.4	4.6	1.4	15.6						
Hem	18.3	1.1	0.4	2.7		15.4	1.1	0.31	3.5						
Unwell	21.2	1.3	0.6	2.6		19.3	1.4	0.50	3.9						

^1^ Age categories were compared to the kitten (<1) category. The *p* value presented is the overall *p* value for the effect of age within the multivariate model. ^2^ Age categories were compared to the young adult category. The *p* value presented is the overall *p* value for the effect of age within the multivariate model. ^3^ Reason for FIV testing categories were compared to the wellness examination category. The *p* value presented is the overall *p* value for the effect of reason for FIV testing within the multivariate model. The dermatological disease category was excluded from analysis due to insufficient sample size. Refer to the text for individual *p* values for each category.

**Table 4:** Comparison of hematology and blood biochemistry results for cats in cohort 1 (*n* = 525; in-house hematology and blood biochemistry testing). Only significant differences are shown (15 out of a total of 37 measurements analysed), with up (↑) and down (↓) arrows signifying significantly different mean values in FIV-infected cats compared to FIV-uninfected cats. Mean values for FIV-infected cats outside of the reference interval (RI) for normal healthy cats are annotated in bold. Only absolute WBC counts (i.e., not relative WBC counts) were evaluated. WBC = white blood cell count, RBC = red blood cell count, Hb = hemoglobin, MCV = mean corpuscular volume, MCHC = mean corpuscular hemoglobin concentration, A:G = albumin to globulin ratio, AST = aspartate aminotransferase, GGT = gamma glutamyl transferase.

Measurement	FIV Status	Predicted Mean	Standard Error	*p* Value	RI
WBC (×10^9^/L)	FIV-uninfected FIV-infected	12.6 9.9 ↓	0.90 1.18	0.006	2.9–17.0
Eosinophils (×10^9^/L)	FIV-uninfected FIV-infected	0.44 0.20 ↓	0.08 0.05	0.024	0.17–1.6
Neutrophils (×10^9^/L)	FIV-uninfected FIV-infected	7.9 6.1 ↓	0.58 0.73	0.013	2.3–10.3
Basophils (×10^9^/L)	FIV-uninfected FIV-infected	0.04 0.03 ↓	0.00 0.00	<0.001	0.01–0.26
Lymphocytes (×10^9^/L)	FIV-uninfected FIV-infected	2.0 1.7 ↓	0.12 0.15	0.047	0.92–6.9
RBC (×10^12^/L)	FIV-uninfected FIV-infected	7.3 6.7 ↓	0.16 0.23	0.013	6.5–12.2
Hb (g/L)	FIV-uninfected FIV-infected	104.5 **95.0** ↓	0.21 0.31	0.006	98.0–162.0
MCV (fL)	FIV-uninfected FIV-infected	42.7 45.0 ↑	0.51 0.77	0.005	35.9–53.1
MCHC (g/L)	FIV-uninfected FIV-infected	338.7 320.7 ↓	0.20 0.33	<0.001	281.0–358.0
Platelet Count (×10^9^/L)	FIV-uninfected FIV-infected	360.4 208.0 ↓	11.00 15.30	<0.001	151–600
Plateletcrit (%) ^1^	FIV-uninfected FIV-infected	0.42 0.35 ↓	0.02 0.04	0.045	0.17–0.86
Total Protein (g/L)	FIV-uninfected FIV-infected	74.2 82.0 ↑	0.75 1.20	<0.001	57.0–89.0
Albumin (g/L)	FIV-uninfected FIV-infected	27.3 26.2 ↓	0.30 0.40	0.023	23.0–39.0
Globulin (g/L)	FIV-uninfected FIV-infected	45.9 **54.7** ↑	0.71 1.15	<0.001	28.0–51.0
A:G ratio	FIV-uninfected FIV-infected	0.62 0.50 ↓	0.01 0.02	<0.001	0.5–1.1

^1^ Plateletcrit (PCT) is the percentage of blood volume filled by platelets and represents the circulating platelet mass. PCT should be interpreted alongside the platelet count and may give an indication of platelet size. E.g., a normal platelet count with a reduced PCT could indicate larger platelets, and vice versa (https://eclinpath.com/hematology/tests/other-platelet-tests/, accessed on 29 June 2022).

**Table 5:** Comparison of hematology and blood biochemistry results for cats in cohort 2 (*n* = 282; external hematology and blood biochemistry testing). Only significant differences are shown (13 out of a total of 39 measurements analysed), with up (↑) and down (↓) arrows signifying significantly different mean values in FIV-infected cats compared to FIV-uninfected cats. RI = reference interval, WBC = white blood cell count, RBC = red blood cell count, Hb = hemoglobin, MCV = mean corpuscular volume, MCHC = mean corpuscular hemoglobin concentration, A:G = albumin to globulin ratio, AST = aspartate aminotransferase, GGT = gamma glutamyl transferase.

Measurement	FIV Status	Predicted Mean	Standard Error	*p* Value	RI
Eosinophils (×10^9^/L)	FIV-uninfected FIV-infected	0.50 0.30 ↓	0.05 0.05	0.005	0.0–1.0
RBC (×10^12^/L)	FIV-uninfected FIV-infected	7.0 6.2 ↓	0.21 0.30	0.016	4.9–10.0
Plasma Protein (g/L)	FIV-uninfected FIV-infected	75.7 84.5 ↑	1.61 3.23	0.016	60.0–90.0
Total Protein (g/L)	FIV-uninfected FIV-infected	74.0 79.4 ↑	1.07 2.09	0.021	60.0–84.0
Globulin (g/L)	FIV-uninfected FIV-infected	45.4 51.8 ↑	1.10 2.13	0.008	31.0–52.0
A:G ratio	FIV-uninfected FIV-infected	0.65 0.52 ↓	0.03 0.04	<0.001	0.5–1.1
AST (U/L)	FIV-uninfected FIV-infected	39.3 53.0	2.67 5.14	0.004	2.0–62.0
Total Bilirubin (µmol/L)	FIV-uninfected FIV-infected	1.6 2.3 ↑	0.17 0.35	0.026	0.0–7.0
GGT (U/L)	FIV-uninfected FIV-infected	0.40 0.77 ↑	0.07 0.14	0.017	0.0–5.0
Calcium (mmol/L)	FIV-uninfected FIV-infected	2.2 2.1 ↓	0.02 0.03	0.01	2.1–2.8
Chloride (mmol/L)	FIV-uninfected FIV-infected	116.2 114.1 ↓	0.40 0.70	0.012	106.0–123.0
Sodium (mmol/L)	FIV-uninfected FIV-infected	150.7 148.4 ↓	0.30 0.60	<0.001	144.0–158.0
Total thyroxine (TT4) (nmol/L)	FIV-uninfected FIV-infected	28.4 23.5 ↓	0.99 1.53	0.012	10.0–60.0

**Table 6:** Comparison of hematology results for cats in cohort 3 (*n* = 425; FIV vaccine efficacy study). Only significant differences are shown (2 out of a total of 13 measurements analysed), with up (↑) and down (↓) arrows signifying significantly different mean values in FIV-infected cats compared to FIV-uninfected cats. Only absolute WBC counts (i.e., not relative WBC counts) were evaluated. RI = reference interval, Hb = hemoglobin.

Measurement	FIV Status	Predicted Mean	Standard Error	*p* Value	RI
Eosinophils (×10^9^/L)	FIV-uninfected FIV-infected	0.41 0.58 ↑	0.05 0.02	0.014	0.2–1.4
Hb (g/L)	FIV-uninfected FIV-infected	130.4 123.9 ↓	3 0.8	0.041	80.0–140.0

## Data Availability

All data presented in this paper are available on request.

## Supplementary Materials

The following supporting information can be downloaded at: https://www.mdpi.com/article/10.3390/v14102177/s1, Supplementary Table S1: FIV point-of-care (PoC) test kits used, Supplementary Table S2: Analysis of possible associations between FIV infection and hematology and biochemistry results of potential clinical significance for cats in cohort 1, Supplementary Table S3: Analysis of possible associations between FIV infection and hematology and biochemistry results of potential clinical significance for cats in cohort 2, Supplementary Table S4: Cause of death (both euthanasia and non-euthanasia) for non-survivor cats in cohort 1 and cohort 2.
