# Supplementary material for: Clinicopathological and Epidemiological Findings in Pet Cats Naturally Infected with Feline Immunodeficiency Virus (FIV) in Australia
Source: Viruses. 2022 Sep 30;14(10):2177. doi: 10.3390/v14102177 (PMC9608632; doi:10.3390/v14102177)
Supplement: Supplementary file 1 [file viruses-14-02177-s001.zip › viruses-1818171-supplementary.pdf]

Supplementary Table S1. FIV point-of-care (PoC) test kits used in cohort 1 (*n* = 525; in-house hematology and biochemistry testing) and cohort 2 (*n* = 282; external hematology and biochemistry testing). All cats in cohort 3 (*n* = 425; FIV vaccine efficacy study) were tested with three different FIV PoC test kits (Witness®, SNAP® Combo and Anigen Rapid®).

Test kit	Cohort 1 (*n* = 525)		Total	Cohort 2 (*n* = 282)		Total
	FIV-uninfected	FIV-infected		FIV-uninfected	FIV-infected	
Witness®	306	98	404	168	47	215
SNAP® Combo	86	33	119	53	12	65
Senspert®	0	1	1	0	1	1
FIV RealPCR™	0	1	1	0	1	1
**TOTAL**	392	133 (25%)	525	221	61 (22%)	282

Supplementary Table S2. Analysis of possible associations between FIV infection and hematology and biochemistry results of potential clinical significance for cats in cohort 1 (*n* = 525; in-house hematology and biochemistry testing). Significant differences ($p < 0.05$) between FIV-infected and FIV-uninfected cats are in bold text. OR = odds ratio, LCI = lower confidence interval, UCI = upper confidence interval.

Variable		FIV-uninfected		FIV-infected					
	Yes/No	*n*	%	*n*	%	OR	LCI	UCI	*p* value
Eosinopenia	No	163	41.6	59	44.4	0.89	0.60	1.3	0.58
(eosinophil count < 0.17 x 10^9/L)	Yes	229	58.4	74	55.6				
**Lymphocytosis**	No	360	91.8	129	97	0.35	0.12	0.98	**0.046**
**(lymphocyte count >6.88 x 109/L)**	Yes	32	8.2	4	3				
Lymphopenia	No	285	72.7	102	76.7	0.81	0.51	1.3	0.37
(lymphocyte count <0.92 x 10^9/L)	Yes	107	27.3	31	23.3				
Neutrophilia	No	272	69.4	100	75.2	0.74	0.48	1.2	0.20
(neutrophil count >10.29 x 10^9/L)	Yes	120	30.6	33	24.8				
Neutropenia	No	277	70.7	91	68.4	1.1	0.76	1.7	0.63
(neutrophil count < 2.3 x 10^9/L)	Yes	115	29.3	42	31.6				
Leukocytosis	No	298	76	111	83.5	0.63	3.8	1.0	0.075
(WBC count >17.02 x 10^9/L)	Yes	94	24	22	16.5				
Leukopenia	No	315	80.4	104	78.2	1.1	0.71	1.8	0.59
(WBC count <2.87 x 10^9/L)	Yes	77	19.6	29	21.8				

Anemia (hematocrit <0.3 L/L)	No	232	59.2	76	57.1	1.1	0.73	1.6	0.68
	Yes	160	40.8	57	42.9				
Low RBC Count (RBC count <6.54 x 10^{12}/L)	No	203	51.8	57	42.9	1.4	0.96	2.1	0.076
	Yes	189	48.2	76	57.1				
**Macrocytosis (MCV >53. fL)**	No	374	95.4	118	88.7	2.6	1.3	5.4	**0.008**
	Yes	18	4.6	15	11.3				
Thrombocytopenia (platelet count <151 x 10^9/L)	No	227	57.9	69	51.9	1.3	0.86	1.9	0.23
	Yes	165	42.1	64	48.1				
Bands suspected (presence of band neutrophils)	No	309	78.8	113	85	0.96	0.62	1.5	0.84
	Yes	83	21.2	20	15				
Hypoalbuminemia (albumin <23 g/L)	No	291	74.2	108	81.2	0.67	0.41	1.1	0.11
	Yes	101	25.8	25	18.8				
**Hyperglobulinemia (globulin >51 g/L)**	No	311	79.3	65	48.9	4.017	2.64	6.104	**<0.001**
	Yes	81	20.7	69	51.1				
**Hyperproteinemia (total protein >89 g/L)**	No	358	91.3	107	80.5	2.559	1.471	4.452	**<0.001**
	Yes	34	8.7	26	19.5				

Supplementary Table S3. Analysis of possible associations between FIV infection and hematology and biochemistry results of potential clinical significance for cats in cohort 2 (n = 282; external hematology and biochemistry testing). Significant differences ($p < 0.05$) between FIV-infected and FIV-uninfected cats are in bold text. OR = odds ratio, LCI = lower confidence interval, UCI = upper confidence interval.

**Variable**		**FIV-uninfected**		**FIV-infected**					
	Yes/No	*n*	%	*N*	%	OR	LCI	UCI	*p* value
Leukocytosis (WBC count >17.02 x 10^9/L)	No	183	82.8	53	86.9	0.73	0.32	1.7	0.45
	Yes	38	17.2	8	13.1				
Leukopenia (WBC count <2.87 x 10^9/L)	No	170	76.9	41	67.2	1.6	0.88	3.0	0.12
	Yes	51	23.1	20	32.8				

Lymphopenia (lymphocyte count <0.92 x 10^9/L)	No	169	76.5	43	70.5	1.4	0.72	2.6	0.34
	Yes	52	23.5	18	29.5				
Neutrophilia (neutrophil count >10.29 x 10^9/L)	No	181	81.9	82	85.2	0.78	0.36	1.7	0.54
	Yes	40	18.1	9	14.8				
Neutropenia (neutrophil count < 2.3 x 10^9/L)	No	185	83.7	46	75.4	1.7	0.85	3.3	0.14
	Yes	36	16.3	15	24.6				
Thrombocytopenia (platelet count <151 x 10^9/L)	No	118	53.4	29	47.5	1.264	0.7166	2.23	0.418
	Yes	103	46.6	32	52.5				
Toxic change in neutrophils (as reported by pathologist)	No	171	77.4	43	80.5	1.4	0.76	2.7	0.27
	Yes	50	22.6	18	29.5				
Activated lymphocytes (as reported by pathologist)	No	181	81.9	55	90.2	0.49	0.20	1.2	0.13
	Yes	40	18.1	6	9.8				
Anemia (hematocrit <0.25 L/L)	No	162	73.3	44	72.1	1.061	0.5638	1.996	0.86
	Yes	59	26,7	17	27.9				
Low RBC Count (RBC <4.9 x 10^{12}/L)	No	167	75.6	46	75.4	1.0	0.52	1.9	0.98
	Yes	54	24.4	15	24.6				
Hypochromia (hemoglobin <97 g/L)	No	158	71.5	41	67.2	1.2	0.67	2.2	0.52
	Yes	63	28.5	20	32.8				
Macrocytosis (MCV >55 fL)	No	195	88.2	48	78.7	2.0	0.97	4.2	0.059
	Yes	26	11.8	13	21.3				
Hypoalbuminemia (albumin <29 g/L)	No	103	46.6	24	39.3	1.3	0.76	2.4	0.31
	Yes	118	53.4	37	60.7				
**Hyperglobulinemia (globulin >52 g/L)**	No	195	**88.2**	40	65.6	3.9	2.0	7.7	**<0.001**
	Yes	26	**11.8**	21	34.4				
**Hyperproteinemia (total protein >84 g/L)**	No	203	91.9	46	75.4	3.7	11.7	7.8	**<0.001**
	Yes	18	8.1	15	24.6				

Supplementary Table S4. Cause of death (both euthanasia and non-euthanasia) for non-survivor cats in cohort 1 and 2 combined (n = 737; in-house hematology and biochemistry testing). Two-tailed Fisher's exact testing was used to calculate p values for each category of death in the cohort according to FIV status. Significant differences ($p < 0.05$) between FIV-infected and FIV-uninfected cats are in bold text. Where multiple disease processes were present, the primary morbidity is displayed below. 'Other' causes of death included urethral obstruction, urinary tract disease, diabetes mellitus, dermatological disease, behavioral conditions, cardiorespiratory disease, client financial constraints, gastrointestinal disease,

hepatobiliary disease, infection, sepsis, trauma and FIV status (two FIV-infected cats were euthanased due to their FIV status at the request of their owners). FIP = feline infectious peritonitis.

**Cause of death**	**Cohort 1 and 2 (*n* = 737)**		***p* value**
	FIV-uninfected	FIV-infected	
Hematological disease	34	10	0.12
Neoplasia	21	17	0.14
Neurological disease	16	2	0.06
Renal disease	24	3	**0.02**
Respiratory disease	4	1	1.00
Suspected FIP	10	0	**0.03**
Severe clinical disease	53	44	**0.001**
Unknown	15	7	1.0
Other	10	8	0.45
**TOTAL**	**187**	**92**	**-**
